# TM9 family proteins control surface targeting of glycine-rich transmembrane domains

**DOI:** 10.1242/jcs.164848

**Published:** 2015-07-01

**Authors:** Jackie Perrin, Marion Le Coadic, Alexandre Vernay, Marco Dias, Navin Gopaldass, Hajer Ouertatani-Sakouhi, Pierre Cosson

**Affiliations:** 1 Department of Cell Physiology and Metabolism, Faculty of Medicine, University of Geneva, 1 rue Michel Servet, Geneva 4 CH-1211, Switzerland; 2 Department of Biochemistry, Sciences II, University of Geneva, 30 quai Ernest-Ansermet, Geneva 4 CH-1211, Switzerland

**Keywords:** Phg1, Secretory pathway, Sorting, TM9 protein, Transmembrane domain

## Abstract

TM9 family proteins (also named Phg1 proteins) have been previously shown to control cell adhesion by determining the cell surface localization of adhesion proteins such as the *Dictyostelium* SibA protein. Here, we show that the glycine-rich transmembrane domain (TMD) of SibA is sufficient to confer Phg1A-dependent surface targeting to a reporter protein. Accordingly, in *Dictyostelium phg1A*-knockout (KO) cells, proteins with glycine-rich TMDs were less efficiently transported out of the endoplasmic reticulum (ER) and to the cell surface. Phg1A, as well as its human ortholog TM9SF4 specifically associated with glycine-rich TMDs. In human cells, genetic inactivation of TM9SF4 resulted in an increased retention of glycine-rich TMDs in the endoplasmic reticulum, whereas TM9SF4 overexpression enhanced their surface localization. The bulk of the TM9SF4 protein was localized in the Golgi complex and a proximity-ligation assay suggested that it might interact with glycine-rich TMDs. Taken together, these results suggest that one of the main roles of TM9 proteins is to serve as intramembrane cargo receptors controlling exocytosis and surface localization of a subset of membrane proteins.

## Abstract (precis)

**Summary:** TM9 proteins facilitate transport to the cell surface of proteins with gylcine-rich transmembrane domains. They might represent a new class of cargo receptors controlling transport in the secretory pathway.

## INTRODUCTION

Localization of membrane receptors at the surface of eukaryotic cells is often essential for them to perform their specific functions, such as cellular adhesion (e.g. integrins), nutrients capture (e.g. transferrin receptor) or signal transduction (e.g. EGF receptor). Along the exocytic pathway, transport of newly synthesized proteins from the endoplasmic reticulum (ER) to the cell surface is tightly controlled to ensure the selective delivery of functional protein complexes (Geva and Schuldiner, 2014). Similarly, selective and regulated endocytosis of surface receptors controls surface residency of membrane proteins, allowing adaptation of cellular physiology to changes in the environment.

Our previous studies have revealed the role of a new class of proteins in the control of cell surface localization of membrane proteins. Both Phg1A (Cornillon et al., 2000), Phg1B (Benghezal et al., 2003) and SadA (Fey et al., 2002) were initially characterized in *Dictyostelium* as membrane proteins necessary for efficient cellular adhesion and phagocytosis. Later studies showed that these three proteins are essential for the efficient surface localization of SibA, a cell surface adhesion molecule with integrin β features (Cornillon et al., 2006; Froquet et al., 2012). The Phg1 family, also referred to as the TM9 family, is characterized by a high degree of sequence similarity, an N-terminal luminal domain preceded by a signal sequence and followed by nine transmembrane domains. There are three members in the Phg1/TM9 family in *Dictyostelium* (Phg1A, Phg1B and Phg1C) (Benghezal et al., 2003), three in *S. cerevisiae* [TMN1 (also known as Emp70), TMN2 and TMN3] (Froquet et al., 2008), three in *Drosophila* (TM9SF2, TM9SF3 and TM9SF4) (Bergeret et al., 2008) and four in humans (TM9SF1 to TM9SF4) (Chluba-de Tapia et al., 1997; Schimmöller et al., 1998). In *Drosophila*, TM9SF2 and TM9SF4, which are highly similar to *Dictyostelium* Phg1A, have also been shown to control phagocytosis by determining the cell surface expression of the phagocytic receptor PGRP-LC (Perrin et al., 2015). Intriguingly, SadA, which is also necessary for efficient cell surface targeting of SibA, exhibits the same general organization as Phg1/TM9 proteins (one signal sequence followed by a large extracellular domain and nine transmembrane domains), but shows no sequence homology to Phg1/TM9 proteins.

Here, we studied the mechanism by which TM9 proteins control surface localization of membrane proteins like SibA. Our results indicate that the transmembrane domain (TMD) of SibA is sufficient to confer Phg1A-dependent surface localization to a reporter protein. This property is due to the presence of glycine residues in the TMD of SibA, to which Phg1A specifically associates. Human TM9SF4 shows the same propensity to associate with glycine-rich TMDs and to ensure their localization at the cell surface. This study suggests that TM9 proteins function as cargo receptors ensuring surface localization of proteins harboring glycine-rich transmembrane domains.

## RESULTS

### Surface localization of glycine-rich TMDs is dependent on Phg1A

**Fig. 1.**
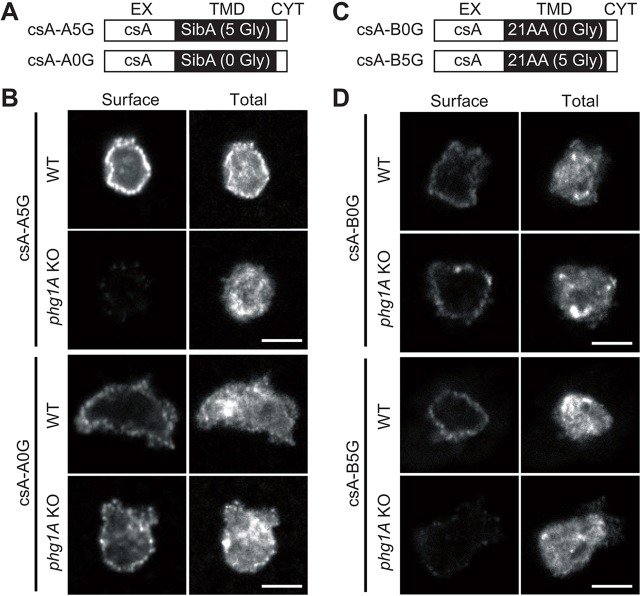
**Phg1A ensures efficient cell surface localization of proteins harboring the SibA glycine-rich TMD.** All pictures were taken with the same confocal microscope (Zeiss LSM700) and the same setting allowing direct comparison. Scale bar: 5 µm. (A) The csA-A fusion proteins are composed of the extracellular domain of csA, the glycine-rich TMD of SibA (csA-A5G) or a mutated form without glycine residues (csA-A0G), and a short cytoplasmic tail (see Table 1). (B) Fusion proteins were labeled before (Surface) and after (Total) permeabilization by immunofluorescence in WT or *phg1A* KO cells, using an antibody specific for the csA extracellular domain. (C) CsA-B fusion proteins are composed of the extracellular domain of csA, a hydrophobic TMD without glycine residues (csA-B0G) or a mutated form with five glycine residues added (csA-B5G), followed by a short cytoplasmic tail (see also Table 1). (D) Fusion proteins were expressed in *Dictyostelium* WT or *phg1A* KO cells and labeled before (Surface) and after (Total) permeabilization by immunofluorescence.

Previous experiments have demonstrated that in *Dictyostelium*, a chimera composed of the extracellular domain of contact site A (csA) fused to the transmembrane and cytosolic domain of SibA is localized at the cell surface less efficiently in *phg1A*-knockout (KO) cells than in wild-type (WT) cells (Froquet et al., 2012). To identify the feature of SibA that confers differential sorting in WT and *phg1A* KO cells, we expressed in these two cell lines a chimeric protein composed of the csA extracellular domain fused to the TMD of SibA and to a very short cytosolic domain (denoted csA-A5G) (Fig. 1A, see also Table 1). The surface localization of the csA fusion proteins was assessed by immunofluorescence. For this, we labeled, with different fluorescent antibodies in non-permeabilized cells, the csA fusion protein exposed at the cell surface and, after permeabilization, the total cellular csA (surface+intracellular) (Fig. 1B). When cells with similar total expression levels of csA were compared, the cell surface localization of csA-A5G was readily detectable in WT cells, but was much lower in *phg1A* KO cells (Fig. 1B). This result indicated that the TMD of SibA is sufficient to render the surface targeting of a reporter membrane protein dependent on Phg1A.

**Table 1.**
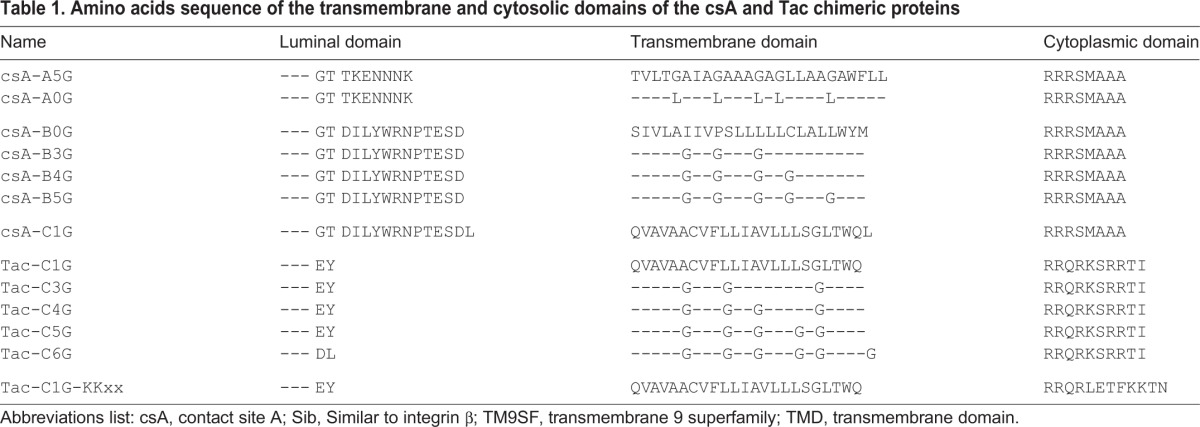
**Amino acids sequence of the transmembrane and cytosolic domains of the csA and Tac chimeric proteins**

The most remarkable feature of the SibA TMD is the presence of five glycine residues, conserved in SibB, SibC, SibD and SibE (Cornillon et al., 2006). When these five residues were mutated to leucine (Fig. 1A; Table 1), the resulting fusion protein (csA-A0G) was targeted to the cell surface as efficiently in WT and *phg1A* KO cells (Fig. 1B). This observation suggests that the multiple glycine residues in the SibA TMD are necessary for Phg1A-dependent surface localization of the protein.

To test this hypothesis further, we assessed the surface localization of csA-B0G, a fusion protein with a 21-residue hydrophobic TMD containing no glycine residues derived from the human CD1b molecule (Mercanti et al., 2010) (Fig. 1C; Table 1). As described previously (Froquet et al., 2012), we observed that this protein is present at the surface of both WT and *phg1A* KO cells at similar levels (Fig. 1D). We then introduced five glycine residues in the TMD of csA-B0G (Fig. 1C; Table 1), and assessed the surface localization of the resulting fusion protein (csA-B5G) in WT and *phg1A* KO cells. CsA-B5G was present at the surface of WT cells, but it was detected at very low levels at the surface of *phg1A* KO cells (Fig. 1D), suggesting that the presence of glycine residues is sufficient to make surface targeting of a TMD dependent on Phg1A.

**Fig. 2.**
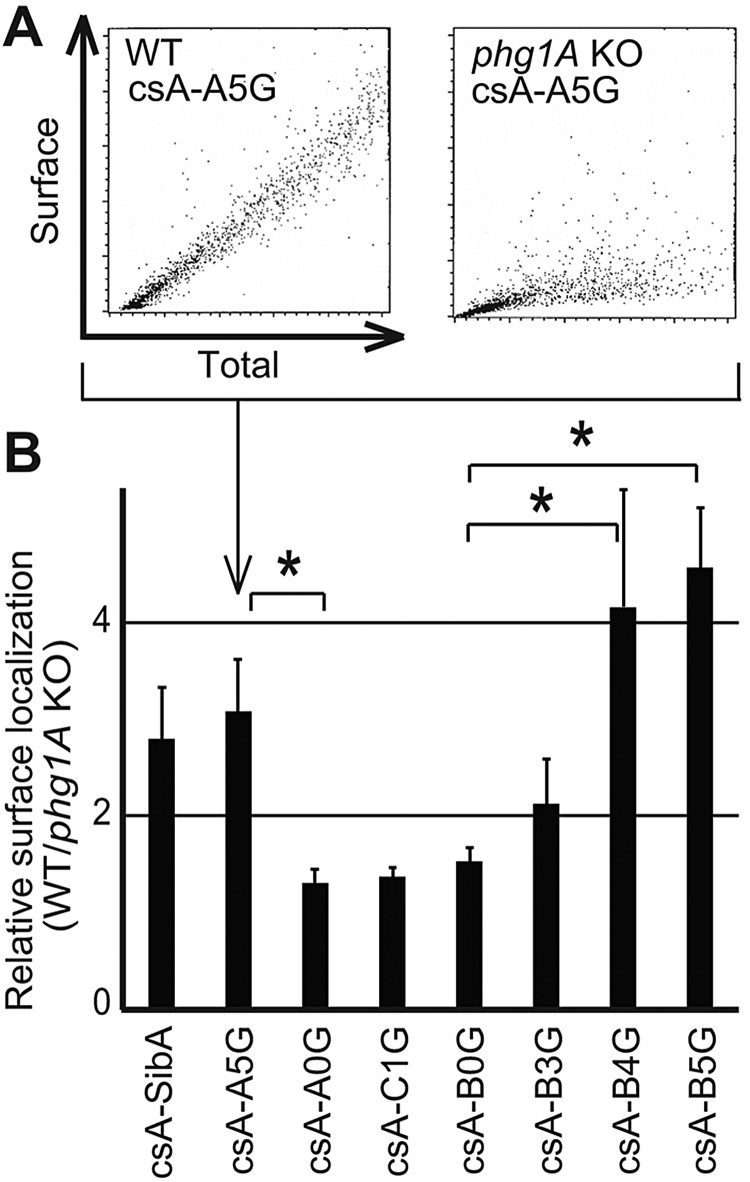
**Efficient surface targeting of glycine-rich TMDs by Phg1A.** (A) Analysis of total expression and cell surface levels of csA-A5G chimeras in WT (left panel) and *phg1A* KO *Dictyostelium* cells (right panel) by flow cytometry. For each cell, the total and the surface levels of csA fusion protein were determined. In order to extrapolate a numeric value from the dot plot analysis, a linear regression was used. (B) We calculated the surface targeting (in arbitrary units) by dividing the slope obtained in A for WT cells and for *phg1A* KO cells. The mean±s.e.m. of at least eight experiments are indicated. *P<0.01 (Student's *t*-test).

In the experiments described above, cells with similar total expression levels were selected, to allow meaningful comparison between different cells. To obtain more quantitative data, we analyzed fluorescently labeled cells by flow cytometry. This technique measures surface and total labeling simultaneously in each individual cell. This analysis revealed the existence of a linear relationship between the total cellular level of csA protein, and the level found at the cell surface (Fig. 2A): higher total levels of csA protein resulted in higher levels at the cell surface. When comparing WT and *phg1A* KO cells, it is also apparent that at equivalent expression levels, a higher amount of csA-A5G is detected at the surface of WT cells than at the surface of *phg1A* KO cells (Fig. 2A). Based on these results, for each chimeric protein, we calculated the relative efficiency of surface targeting in WT and *phg1A* KO cells (surface csA in WT cells divided by surface csA in *phg1A* KO cells). Accordingly, csA-A5G, which exhibits the glycine-rich TMD of SibA, was targeted three times more efficiently to the surface of WT cells than to the surface of *phg1A* KO cells (Fig. 2B). By contrast, three different fusion proteins (csA-A0G, csA-B0G and csA-C1G) with a hydrophobic TMD exhibiting at most one glycine residue were localized as efficiently to the surface of WT and of *phg1A* KO cells (Fig. 2B). Addition of five glycine residues to the csA-B0G TMD (csA-B5G) made it dependent on Phg1A for cell surface targeting, whereas surface localization of proteins with three or four glycine residues (csA-B3G and csA-B4G) was partially dependent on Phg1A (Fig. 2B).

**Fig. 3.**
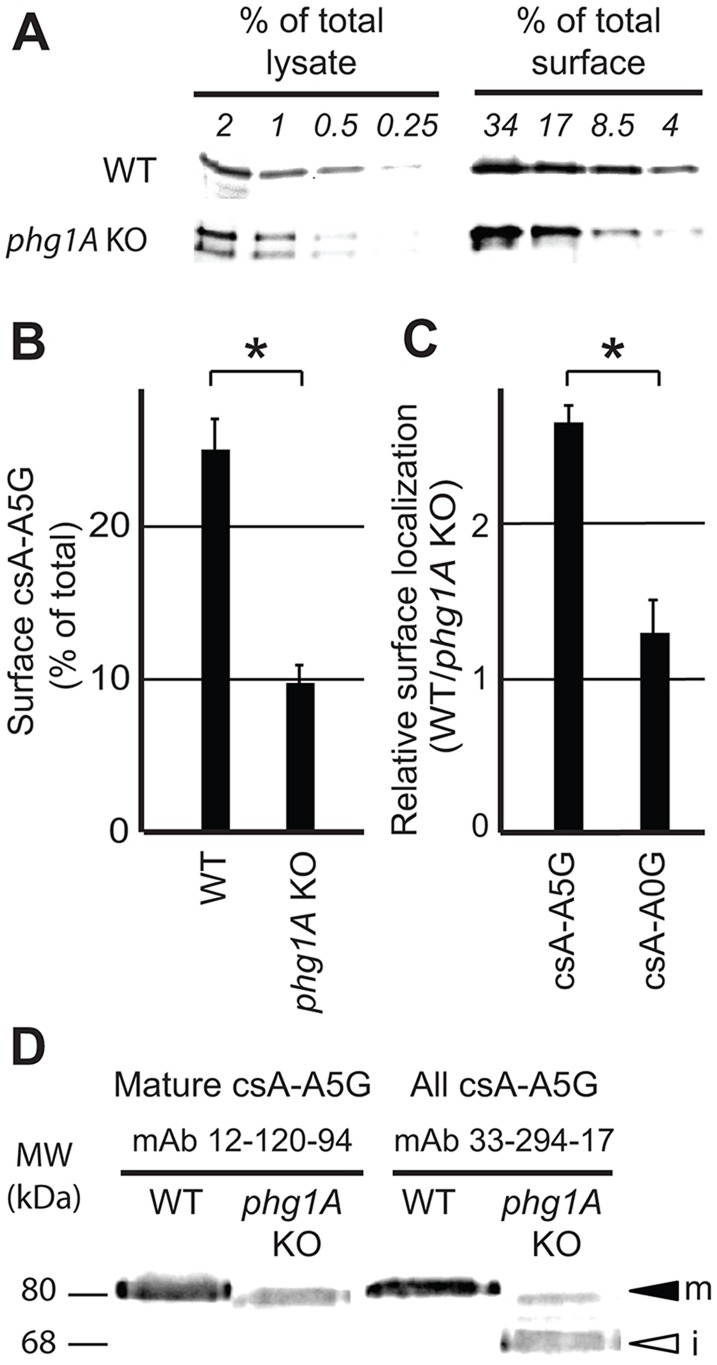
**Biochemical analysis confirms differential surface localization of glycine-rich TMDs in WT versus *phg1A* KO cells.** (A) Following cell surface biotinylation, surface csA-A5G fusion protein was purified on neutravidin beads, and its level compared to the total cellular level by western blotting after serial dilution. The percentage of total or surface proteins loaded on each lane is indicated. Based on these results, the percentage of csA-A5G fusion protein present at the cell surface was determined in WT (20.5% of total) and *phg1A* KO cells (7% of total) (see supplementary material Fig. S1A for details of the quantification). (B) The mean±s.e.m. of three independent experiments as described in A was determined. CsA-A5G was significantly more abundant at the surface of WT cells than at the surface of *phg1A* KO cells. (C) Relative targeting of each fusion protein in WT versus *phg1A* KO cells was obtained by dividing the percentage of surface csA in WT cells by that in *phg1A* KO cells (e.g. 20.5/7.0 in A). The mean±s.e.m. of at least three independent experiments are indicated. Cell surface localization of csA-A5G, but not of csA-A0G, was decreased in *phg1A* KO cells relative to WT cells. *P<0.01 (Student's *t*-test). (D) Export of csA-A5G out of the ER is affected in *phg1A* KO cells. Cellular lysates of WT or *phg1A* KO *Dictyostelium* cells were analyzed by western blotting using antibody 12-120-94, which detects only mature csA, and antibody 33-294-17, which detects both mature and immature csA. The mature csA-A5G (m) exhibited a molecular mass of 80 kDa, whereas the low-molecular-mass form (68 kDa) detected in *phg1A* KO cells corresponded to the immature ER form (i).

To verify the validity of these observations, the presence of csA-A5G and csA-A0G at the surface of WT and *phg1A* KO cells was also assessed biochemically. For this, cell surface proteins were labeled with biotin and purified on neutravidin beads. The amount of csA in the cell (total cell lysate) and at the surface (biotinylated proteins) was then assessed by western blotting, using antibodies specific for the csA extracellular domain. One representative experiment is shown (Fig. 3A), which indicated that 20.5% of csA-A5G was present at the surface of WT cells, but only 7.0% at the surface of *phg1A* KO cells (see supplementary material Fig. S1A for details of the quantification procedure). Quantification of three independent experiments confirmed that csA-A5G was significantly more abundant at the surface of WT cells than at the surface of *phg1A* KO cells (Fig. 3B). Based on these results, we calculated the relative surface targeting in WT and *phg1A* KO cells and confirmed that surface localization of csA-A5G was dependent on Phg1A (2.5 times more surface csA in WT than in *phg1A* KO cells) (Fig. 3C). By contrast, surface targeting of csA-A0G was similar in WT and in *phg1A* KO cells (equivalent surface levels of csA in WT and *phg1A* KO cells) (Fig. 3C).

Taken together, these results demonstrate that efficient surface targeting of a membrane protein exhibiting a glycine-rich TMD requires the presence of the Phg1A protein.

### Efficient ER exit of proteins with glycine-rich TMDs is dependent on Phg1A

The csA protein is inserted co-translationally in the ER, where it is rapidly converted into a glycosylated 68-kDa protein. It is then further modified as it passes through the Golgi complex, to reach its final molecular mass of 80 kDa (Hohmann et al., 1985). For most csA fusion proteins, at steady state, an 80-kDa form, presumably corresponding to the mature form was predominant, and the immature 68-kDa form was hardly detectable (see for example supplementary material Fig. S1A). By contrast, as can be seen in Fig. 3A, for csA-A5G, the 68-kDa form, presumably corresponding to the immature form of csA, was abundant in *phg1A* KO cells. Quantification of several independent experiments revealed that 17±8% of csA-A5G is immature in WT cells versus 57±2% in *phg1A* KO cells (mean±s.e.m.; *P*<0.001; *n*=5). To verify that the lower-molecular-mass csA-A5G protein detected in *phg1A* KO cells was truly an immature form, we used a monoclonal antibody (12-120-94) which recognizes specifically the mature form of csA (Ochiai et al., 1982). As expected, only the 80-kDa form of csA-A5G was detected by the 12-120-94 antibody (Fig. 3D), confirming that the lower-molecular-mass form corresponds to an immature protein present in the ER.

A low level of mature csA fusion protein might indicate that the protein is retained in the ER, or that it is rapidly degraded after exiting the ER. To distinguish between these two possibilities, we measured the stability of csA fusion proteins in cells where protein synthesis was inhibited. These experiments revealed no significant difference between the stability of csA-A5G or csA-A0G in WT versus *phg1A* KO cells (supplementary material Fig. S1B,C). Taken together, these experiments indicate that csA-A5G is transported inefficiently out of the ER in *phg1A* KO cells, suggesting that Phg1A facilitates transport of glycine-rich TMDs at early stages of the secretory pathway.

### Phg1A specifically interacts with glycine-rich TMDs

**Fig. 4.**
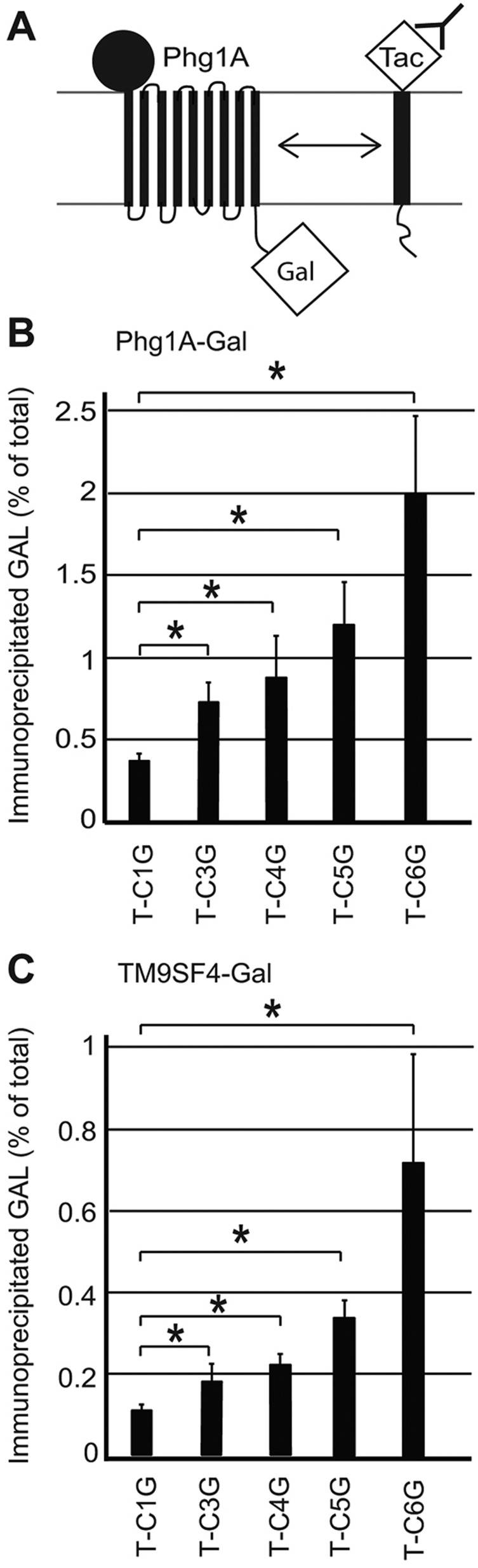
***Dictyostelium* Phg1A and human TM9SF4 associate preferentially with glycine-rich TMDs.** (A) To reveal a putative association of Phg1A with glycine-rich TMDs, COS7 cells were co-transfected with plasmids encoding the Phg1A protein fused to β-galactosidase (Gal) and Tac fusion proteins fused to various TMDs. Tac fusion proteins were immunoprecipitated and the amount of co-precipitated β-galactosidase activity assessed to reveal the degree of association with Phg1A. (B) Phg1A–Gal was co-expressed with T-C1G (one glycine in the TMD) or with Tac mutants with three (T-C3G), four (T-C4G), five (T-C5G) or six (T-C6G) glycine residues in their TMD. Addition of glycine residues in the TMD of Tac gradually increased its interaction with Ph1A–Gal. The mean±s.e.m. of at least six experiments are indicated. (C) Interaction between human TM9SF4 and glycine-rich TMDs was determined as described in B. A specific interaction was detected between TM9SF4 and glycine-rich TMDs. The mean±s.e.m. of at least eight experiments are indicated. *P<0.05 (Student’s *t*-test).

Previous attempts to detect an interaction between SibA and Phg1A have been unsuccessful (Froquet et al., 2012). However, a transient or low-affinity interaction would easily escape detection. Recently, the *Drosophila* TM9SF2 and TM9SF4 have been shown to control the cell surface expression of PGRP-LC, and to interact with it (Perrin et al., 2015). To establish more finely whether Phg1A associates specifically with glycine-rich TMDs, we used a system previously designed to assess the interactions between transmembrane proteins (Cosson et al., 1991): the *phg1A* gene was fused to the coding sequence of β-galactosidase (Phg1A-Gal) in a vector allowing transient expression in transfected COS7 cells. A reporter protein, the α-subunit of the interleukin-2 receptor (Tac antigen) was engineered to introduce three to six glycine residues in its TMD (T-C3G, T-C4G, T-C5G and T-C6G, see Table 1), and these various Tac fusion proteins were co-expressed with Phg1A-Gal (Fig. 4A). After immunoprecipitation of the Tac antigen, the amount of co-precipitated β-galactosidase was measured and compared to the amount of total cellular β-galactosidase. Only one glycine residue is present in the Tac TMD (T-C1G), and less than 0.5% of the β-galactosidase was co-precipitated with T-C1G (Fig. 4B). The amount of co-precipitated β-galactosidase increased gradually when three, four, five or six glycine residues were introduced (Fig. 4B). Interestingly, TM9SF4, the human ortholog of Phg1A, showed a virtually identical pattern of association with glycine-rich TMDs (Fig. 4C), suggesting that it is also capable of interacting specifically with glycine-rich TMDs, and that the role of TM9 proteins in intracellular sorting might be conserved from *Dictyostelium* to human cells.

### TM9SF4 escorts glycine-rich TMDs to the cell surface in human cells

To assess the role of TM9SF4 in intracellular transport in human cells, we created three independent targeted CRISPR/Cas9-knockout cell lines by inactivating the *TM9SF4* gene in HEK293T cells (supplementary material Fig. S2A). In these three knockout cell lines, a fraction of the cells appeared multinucleated (supplementary material Fig. S2B), an observation similar to that made in *Dictyostelium*, where inactivation of two members of the TM9 family resulted in a cytokinesis defect (Benghezal et al., 2003). In these *TM9SF4* KO cells, as well as in cells overexpressing a Flag-tagged TM9SF4, the general organization of the ER, as visualized by expressing an ER-targeted fluorescent protein (ER–YFP), appeared unperturbed (supplementary material Fig. S2C). Similarly, the general organization of the Golgi complex visualized with an antibody against giantin (also known as GOLGB1) was not altered (supplementary material Fig. S2D).

We next expressed in WT or *TM9SF4* KO cells a series of chimeric proteins exhibiting an increasing number of glycine residues in their TMDs (Table 1), and we determined by immunofluorescence their intracellular localization. Fewer than five glycine residues allowed massive localization of Tac at the cell surface. By contrast, the presence of five or six glycine residues in the TMD (T-C5G or T-C6G, respectively) reduced strongly their surface localization (supplementary material Fig. S3A). The intracellular T-C5G and T-C6G were found mostly in the ER, as can be deduced from their perinuclear localization, and as assessed by colocalization with a marker of the ER (ER–YFP) (supplementary material Fig. S3B). This result indicates that glycine residues in the TMD of membrane proteins can affect their surface targeting in mammalian cells.

**Fig. 5.**
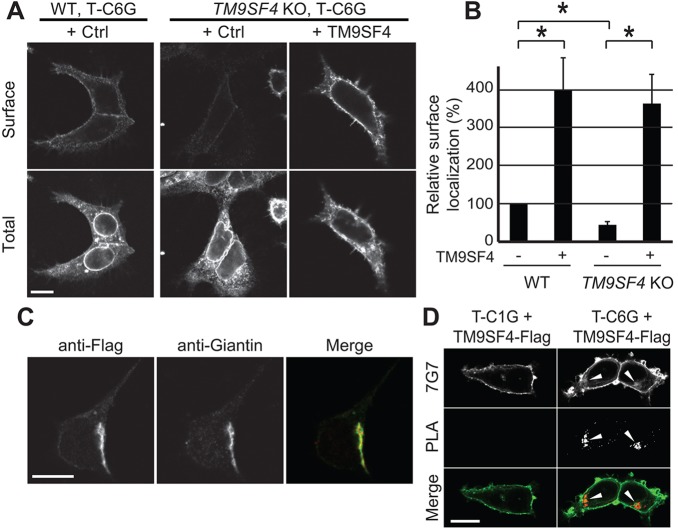
**TM9SF4 controls surface localization of glycine-rich TMDs in human cells.** (A) A Tac protein with six glycine residues in its TMD (T-C6G) was expressed in parental (WT) or *TM9SF4* KO mammalian HEK293T cells. The protein was labeled before (Surface) and after (Total) cell permeabilization. Large amounts of T-C6G were detected in the ER in both cell types, as evidenced by the clearly visible nuclear envelope. Lower cell surface levels were observed in *TM9SF4* KO cells than in parental cells. Moreover, overexpression of TM9SF4 (+TM9SF4) increased strongly cell surface levels of T-C6G. All the pictures presented here were taken sequentially with identical settings. (B) Quantification of the surface targeting of T-C6G in parental and *TM9SF4* KO HEK293T cells, and in cells overexpressing TM9SF4. The mean±s.e.m. of four independent experiments are presented. *P<0.01 (Student's *t-*test). (C) A Flag-tagged version of TM9SF4 was expressed in HEK293T cells, and detected by immunofluorescence. It colocalized with giantin, a marker of the Golgi complex. (D) T-C1G (upper panel) or T-C6G (lower panel) were co-expressed with a Flag-tagged version of TM9SF4 in HEK293T WT cells. A proximity ligation assay (PLA) revealed a specific signal in the Golgi complex when T-C6G and TM9SF4–Flag were co-expressed (arrowheads). Scale bars: 10 µm.

We focused our subsequent studies on the transport of T-C6G to the cell surface, because this protein associated most efficiently with TM9SF4 (see Fig. 4C). The intracellular localization of T-C6G was assessed in WT or *TM9SF4* KO cells expressing similar levels of fusion protein. In both cell types, the majority of T-C6G was found in the ER (Fig. 5A; supplementary material Fig. S3B). The surface level of T-C6G was weak in WT cells, and appeared even weaker in *TM9SF4* KO cells (Fig. 5A). To quantify these observations, the surface level of T-C6G was determined relative to the total expression level in individual cells, and it was indeed significantly reduced in *TM9SF4* KO cells compared to parental cells (Fig. 5B). Conversely, when TM9SF4 was overexpressed in parental and in *TM9SF4* KO, cell surface targeting of T-C6G was significantly increased (Fig. 5B). Taken together, these observations suggest that, like *Dictyostelium* Phg1A, human TM9SF4 associates specifically with glycine-rich TMDs and ensures that they are transported to the cell surface. Interestingly, neither genetic inactivation of *TM9SF4*, nor overexpression of TM9SF4 affected the ER localization of a Tac chimeric protein that was retained in the ER by virtue of a cytosolic dilysine motif (supplementary material Fig. S3C). This result indicates that no general defect in ER retention is observed in *TM9SF4* KO cells or in cells overexpressing TM9SF4. Similarly, we did not detect the induction of an ER stress response in *TM9SF4* KO cells or in cells overexpressing TM9SF4 (supplementary material Fig. S4). Thus, TM9SF4 controls the intracellular transport of a small set of proteins, without altering the general organization and function of the ER.

In order to assess further the function of the TM9SF4 protein, we expressed a tagged version of TM9SF4. Overexpression of the Flag-tagged TM9SF4 also increased cell surface expression of T-C6G (data not shown), indicating that it functions like the wild-type TM9SF4 protein. By assessing immunofluorescence, we found that Flag-tagged TM9SF4 was present in a juxtanuclear compartment, where it was colocalized with giantin (also known as ???), a marker of the Golgi complex (Fig. 5C).

Given that TM9SF4 is present in the Golgi complex, whereas the majority of the T-C6G was found in the ER (in WT cells) or at the cell surface (in cells overexpressing TM9SF4), we tested directly whether a fraction of T-C6G colocalized with TM9SF4. For this, we used a proximity-ligation assay, which detects very close proximity between two proteins (below 30 nm) (Söderberg et al., 2006). A small fraction of T-C6G was detected in close proximity with TM9SF4 (Fig. 5D), suggesting that at any given time a fraction of T-C6G is colocalized with TM9SF4 in the Golgi complex, where the two proteins are in close proximity and might interact.

**Fig. 6.**
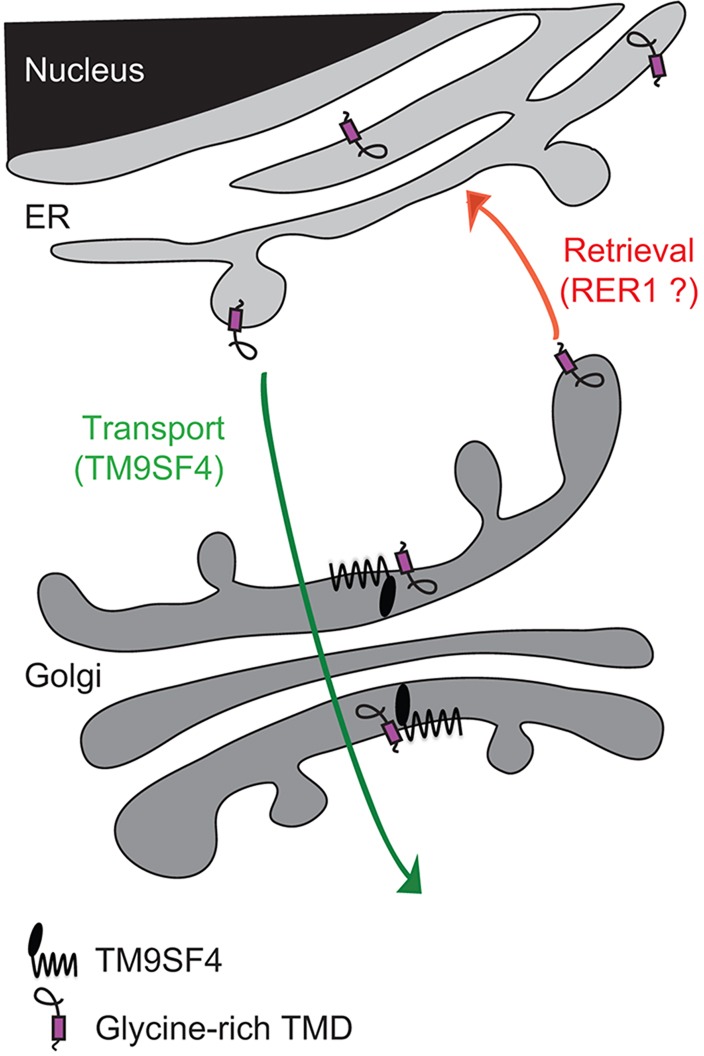
**A speculative model of the role of TM9SF4 in the sorting of glycine-rich TMDs.** Glycine-rich TMDs can ensure ER localization of a membrane protein, presumably by interacting with the well-characterized Rer1 retrieval receptor. Human TM9SF4 is localized in the Golgi complex and associates specifically with glycine-rich TMDs to ensure their transport to the cell surface.

Overall our results suggest that TM9SF4 interacts with glycine-rich TMDs and allows them to be transported through the Golgi complex and to the cell surface (Fig. 6).

## DISCUSSION

Numerous studies have established that TMDs can be essential elements in controlling intracellular transport of individual membrane proteins (reviewed in Cosson et al., 2013). Length and hydrophilicity are key features defining the influence of a given TMD on intracellular sorting, but its exact amino acid sequence can also be a determining factor (Sharpe et al., 2010). Molecular mechanisms ensuring differential sorting of TMDs have been best studied in the early steps of the secretory pathway, where short or hydrophilic TMDs are retained in the ER. Specific ER localization of short or hydrophilic TMDs might result from an inefficient exit out of the ER (Ronchi et al., 2008), coupled to a continuous retrieval of escaped proteins back to the ER after their recognition in the cis-Golgi by the Rer1 receptor (Sato et al., 2001). By contrast, proteins with long hydrophobic TMDs are recognized by Erv14 in the ER and efficiently packaged in COPII-coated secretory vesicles (Herzig et al., 2012). Recognition of TMDs by specific cargo receptors, like Erv14, or retrieval receptors, like Rer1, might control their intracellular transport at each transport step in eukaryotic cells. It is worth noting that, to date, virtually all our knowledge of the molecular mechanisms ensuring sorting of TMDs is based on results obtained in yeast cells, and potential cargo or retrieval receptors have not been characterized to the same extent in mammalian cells.

Our results suggest that Phg1A and TM9SF4 act as a cargo receptor enabling specifically transport of glycine-rich TMDs to the cell surface, both in *Dictyostelium* and in human cells. Another interpretation, not mutually exclusive with the first one, is that Phg1A and TM9SF4 might act as a chaperone facilitating folding of glycine-rich TMDs and facilitating their transport along the secretory pathway. These two interpretations would account for the observation that surface localization of proteins with glycine-rich TMDs is reduced by the genetic inactivation of Phg1A or TM9SF4, and increased by TM9SF4 overexpression in mammalian cells. Both Phg1A and TM9SF4 show a specific propensity to associate with glycine-rich TMDs. More specifically, in order to assist transport of glycine-rich TMDs, Phg1A and TM9SF4 would need to associate with them in the early secretory pathway and release them in later compartments (Golgi complex or cell surface). In agreement with this proposal, TM9SF4 is localized mostly in the Golgi complex, and a proximity-ligation assay suggests that it might interact with glycine-rich TMDs in this compartment. Our results do not exclude the possibility that Phg1A or TM9SF4, or other TM9 proteins, might also control transport of proteins at other steps of intracellular transport. Indeed, there have been scattered reports that TM9 proteins are present both in the Golgi complex and in endocytic compartments in various species (discussed in Pruvot et al., 2010), but the functional significance of these observations remains to be firmly established.

Glycine-rich TMDs are not exceptional in eukaryotic cells. Like hydrophilic residues, glycine residues allow specific interactions between TMDs, thus driving the formation of homo- or hetero-oligomeric complexes and playing a key role in the function of many membrane receptors (Fink et al., 2012). For example, GxxxG motifs, where two glycine residues are placed on the same face of the TMD helix participate in the dimerization of the α and β subunits of some integrin molecules (Kim et al., 2009). Higher numbers of glycine residues on the same face of the TMD helix also drive the homo-oligomerization of glycophorin A, or the hetero-oligomerization of class II major histocompatibility complex proteins (Cosson and Bonifacino, 1992; Lemmon and Engelman, 1994). In the human amyloid precursor protein, at least three glycine residues in the TMD have been implicated in formation of homodimers, thus controlling the intracellular fate of the protein and the generation of amyloid peptides (Kienlen-Campard et al., 2008; Munter et al., 2007). Our results suggest that TM9 proteins might participate in the intracellular transport of these proteins. In SibA itself, glycine residues are mostly placed on the same face of the TMD helix (Cornillon et al., 2006), a disposition favoring interactions with other glycine-rich TMDs. Several TMDs of TM9 proteins exhibit GxxG or GxxxG motifs (notably TMDs 4 and 9 in Phg1A and TM9SF4) and could thus directly associate with glycine-rich TMDs to assist their transport. In addition to SibA, the intracellular sorting of two proteins so far has been shown to depend on TM9 proteins: the *Dictyostelium* Kil1 protein and the *Drosophila* PGRP-LC receptor. Both Kil1 and PGRP-LC are type II transmembrane proteins, and their TMDs do not exhibit multiple glycine residues. Thus, it is possible that the specificity of TM9 proteins is not limited to glycine-rich TMDs.

The finding that TM9 proteins participate in the intracellular transport of a subset of proteins with glycine-rich TMDs might account for the variety of phenotypes observed in cells with altered levels of TM9 proteins. A complete understanding of the proteins placed under the control of TM9 proteins might be essential to fully understand their role in intracellular transport. In *D. discoideum*, where the function of TM9 proteins has been most extensively studied, *phg1A* KO cells have been shown to be defective in cellular adhesion (due to depletion of surface SibA) (Froquet et al., 2012), in intracellular bacterial killing (due to depletion of Kil1, a Golgi sulfo-transferase) (Benghezal et al., 2006; Le Coadic et al., 2013), and in intracellular targeting of lysosomal enzymes (Froquet et al., 2008). Similarly in *S. cerevisiae*, *D. melanogaster* and human cells, a variety of functions (adhesion, phagocytosis, intracellular transport, autophagy, nutrient sensing etc.) are known to be altered by disruption or overexpression of TM9 proteins. It is also worth noting that the genetic alterations described in this study did not generate all-or-none effects: typically, genetic inactivation of Phg1A or TM9SF4 resulted in a three-fold decrease of the surface targeting of glycine-rich TMDs. Previous studies have shown that at least three proteins facilitate surface targeting of SibA in *Dictyostelium*: Phg1A, Phg1B and SadA. Functional redundancy might thus attenuate the effect of a single gene inactivation. In the future, it will be interesting to determine whether other TM9 proteins interact specifically with different subsets of TMDs, and if they act at the same steps of intracellular transport.

## MATERIALS AND METHODS

### Cell lines and media

*Dictyostelium discoideum* DH1-10 cells (Cornillon et al., 2000) were grown in HL5 medium at 23°C and are referred to as wild-type (WT). *Phg1A* KO cells were as described previously (Benghezal et al., 2003). These two strains were modified by introducing a plasmid encoding the Rep protein, allowing replication of plasmids bearing the Ddp2 origin of replication (Shammat and Welker, 1999). Cells were diluted twice a week to maintain cultures at a maximal density of 1.5×10^6^ cells/ml.

### Surface targeting of csA chimeric proteins in *Dictyostelium*

The plasmid allowing the expression of a csA–SibA chimera was as described previously (Froquet et al., 2012). In this study, all csA chimeric proteins were obtained similarly by subcloning PCR fragments in the csA-0 plasmid digested with *Kpn*I and *Xba*I. The sequence of the TMD of each construct is shown in Table 1. Plasmids were transfected in WT or *phg1A* KO cells as described previously (Alibaud et al., 2003). Cells expressing csA chimeric proteins were selected and maintained in HL5 supplemented with G418 (12.5 µg/ml).

To analyze cell surface levels of csA fusion proteins in *Dictyostelium*, 0.5×10^6^ cells were allowed to attach on a glass coverslip for 10 min at room temperature in phosphate buffer (2 mM Na_2_HPO_4_, 14.7 mM KH_2_PO_4_ pH 6.0 supplemented with 100 mM sorbitol, 100 µM CaCl_2_ and 0.5% HL5) (Smith et al., 2010). Cells were washed in phosphate buffer, labeled for 2 min with a monoclonal antibody (41-71-21) against native csA (Bertholdt et al., 1985), washed in phosphate buffer and fixed for 10 min in phosphate buffer containing 4% paraformaldehyde. After washing in PBS containing 20 mM NH_4_Cl, and in PBS containing 0.2% BSA (PBS-BSA), cells were incubated for 20 min with an Alexa-Fluor-647-coupled anti-mouse-IgG antibody in PBS-BSA. After a washing step in PBS-BSA, cells were permeabilized with Triton X-100 (0.07% in PBS for 2 min), and washed in PBS-BSA. This procedure is optimal for staining of cell surface proteins (Vernay and Cosson, 2013). The whole cellular csA content was labeled with the 41-71-21 antibody in PBS-BSA for 20 min, then cells were washed twice in PBS-BSA and incubated for 20 min with an Alexa-Fluor-488-coupled anti-mouse-IgG antibody in PBS-BSA. Finally, cells were washed twice in PBS-BSA, once in PBS and mounted in Mowiol for visualization in an LSM700 confocal microscope (Zeiss), or scrapped from the coverslip and resuspended in PBS for flow cytometry analysis.

To label cell surface proteins with biotin, 30×10^6^ cells were harvested and washed in 10 ml SB-Sorbitol (phosphate buffer Na_2_HPO_4_-KH_2_PO_4_ 17 mM pH 6.0 containing 120 mM sorbitol). The pellet was resuspended in 2 ml of SB-Sorbitol pH 8.0 containing 1 mg of NHS-SS-biotin (Pierce) and incubated on ice for 10 min. Cells were harvested and resuspended in 10 ml PBS supplemented with 100 mM Glycine for 5 min on ice. Biotinylated cells were washed four times in 10 ml SB-Sorbitol pH 6.0 and an aliquot of 500 µl was collected to analyze the total amount of csA. Cells were then lysed for 15 min in 1 ml RIPA buffer [50 mM Tris-HCl pH 7.4, 150 mM NaCl, 1% Triton X-100, 1% sodium deoxycholate, 0.1% SDS and proteases inhibitors (20 µg/ml leupeptin, 10 µg/ml aprotinin, 18 µg/ml PMSF and 18 µg/ml iodoacetic acid)] and the lysate cleared by centrifugation (5 min, 9300 ***g***, 4°C). After centrifugation, 900 µl of the supernatant was incubated with neutravidin beads overnight on a rotating wheel at 4°C. Beads were washed twice with 1 ml of RIPA buffer, incubated for 15 min at 4°C in 1 ml RIPA buffer, incubated in 1 ml of urea 6 M for 15 min at 4°C, and washed three times with 1 ml RIPA buffer. Biotinylated surface proteins were eluted in 50 µl sample buffer for 15 min at room temperature followed by 5 min at 60°C. 20 µl of sample were loaded for each dilution on a 9% SDS-PAGE gel, transferred onto nitrocellulose, and revealed with a mouse anti-csA monoclonal antibody 33-294-17 (Bertholdt et al., 1985). Serial dilutions of the precipitated material were analyzed to quantitatively assess the amounts of csA proteins.

### Tac fusion proteins and association assays

We used a pCDM8-based vector containing the coding sequence of the α chain of the interleukin-2 receptor (Tac) and a *Bgl*II site in the membrane-proximal area (Cosson et al., 1991). The indicated constructs were obtained by inserting the sequence coding for the TMD of interest (see Table 1) in this vector digested with *Bgl*II and *Xba*I. Plasmids were propagated in bacteria MC1061/P3 after chemical transformation and selection on LB agar plates containing ampicillin 12.5 μg/ml and tetracyclin 7.5 μg/ml.

COS7 cells were co-transfected with Tac constructs and a human-codon-optimized version of Phg1A fused to β-galactosidase. Cells were washed in PBS, lysed for 15 min at 4°C in lysis buffer [PBS containing 0.5% Triton X-100 and protease inhibitors (leupeptin 20 µg/ml, aprotinin 10 µg/ml, PMSF 18 µg/ml and iodoacetic acid 18 µg/ml)], then centrifuged for 15 min at 4°C (10,000 ***g***). The supernatant was collected and is referred to as total lysate; an aliquot of 10 µl was kept for analysis of the total amount of β-galactosidase activity. Total lysate was incubated for 1 h at 4°C with protein-A–agarose beads previously coated with an anti-Tac mouse antibody (7G7) (Rubin et al., 1985), then the beads were washed five times with PBS with 0.1% Triton X-100. The β-galactosidase activity was assessed in the total lysate and in the immunoprecipitated sample. We used the substrate Chlorophenol Red-β-D-galactopyranoside and quantified the product of the reaction by absorbance at 600 nm. To assess association of Tac proteins with TM9SF4, a similar procedure was followed, but using HeLa cells.

### Analysis of human cells

HEK293T cells expressing Tac proteins were washed in ice-cold DMEM and the Tac protein at the cell surface was labeled for 15 min with 7G7 antibody (Rubin et al., 1985) in DMEM at 4°C. Cells were then washed in DMEM at 4°C, fixed for 10 min in PBS containing 4% paraformaldehyde, washed in PBS containing 20 mM NH_4_Cl and incubated for 30 min with an Alexa-Fluor-647-coupled anti-mouse-IgG antibody (Life Technologies, A21235) in PBS-BSA at room temperature. Cells were washed three times with PBS-BSA, permeabilized for 10 min in PBS containing 0.2% saponin, washed with PBS-BSA and the intracellular Tac was labeled with 7G7 antibody in PBS-BSA for 30 min. When indicated, samples were also incubated with a rabbit anti-Flag antibody (Sigma, F7425) or a mouse anti-Giantin antibody (Nizak et al., 2003). Cells were washed three times in PBS-BSA and incubated for 30 min with an Alexa- Fluor-488-coupled anti-mouse-IgG antibody (Life Technologies, A11029), an Alexa-Fluor-488-coupled anti-rabbit-IgG antibody (Life Technologies, A11034), or an Alexa-Fluor-647-coupled anti-mouse-IgG antibody (Life Technologies, A21235). Cells were washed again three times with PBS-BSA, once with PBS and mounted in Mowiol. When indicated, the endoplasmic reticulum was identified by expressing YFP-KDEL (ER-YFP, a kind gift of Nicolas Demaurex, University of Geneva, Switzerland).

Surface labeling was quantified with ImageJ software (http://rsb.info.nih.gov/ij/). For each cell, three lines were drawn perpendicular to the cell surface and the height of the peak of fluorescence was averaged, which represented the surface labeling (in arbitrary units). Three lines of defined length were drawn inside the cell, the area under the curve was calculated and averaged, which represented the intracellular labeling (in arbitrary units). The surface:intracellular ratio was then calculated for each individual cell. In each independent experiment, at least 20 cells were quantified.

We used the previously described CRISPR/Cas9 method (Mali et al., 2013) to generate *TM9SF4-*knockout HEK293T cells. In the plasmid purchased from Sigma-Aldrich, the 20-nt guide RNA was designed towards the signal sequence, in the second exon of *TM9SF4* (CCCTGA*TGTGTGAAACAAGCGC, where * is the cutting site of the Cas9 nuclease). 2 µg of plasmid were transfected into HEK293T cells using Lipofectamine 2000 (Life Technologies, 11668-027). After 2 days, GFP-positive cells were sorted by flow cytometry, cloned and allowed to grow, before genomic DNA extraction (QIAamp DNA Blood Mini Kit, Qiagen, 51104). 54 individual clones were screened by PCR using the following primers (primer 1, 5′-CGGTTTTGGAGAAACTTGTAGG-3′; primer 2, 5′-CTTGTTTCACACATCAGGGAG-3′; primer 3, 5′-CTCCCTGATGTGTGAAACAAG-3′; primer 4, 5′-CCAAGGAAAAGAGACGTTCAC-3′ – primers 2 and 3 anneal at the expected site of mutagenesis and should not anneal anymore if mutations occur, and primer 1 and 4 anneal 500 bp on either sides of the Cas9 nuclease cutting site). Pairs of primers are used as follows: 1+2, 3+4 and 1+4. At least one of the PCR amplification was defective in 13 clones, and the genomic region was amplified with primers 1 and 4 and sequenced. Only clones with mutations inducing a frame shift in both alleles were kept.

To perform a proximity ligation assay, HEK293T WT cells were transfected with a Flag-tagged version of TM9SF4 and T-C1G or T-C6G. Cells were fixed, permeabilized, and incubated with the corresponding primary antibodies as described above. The proximity ligation assay was performed according to the manufacturer's protocol (Sigma, DUO92101).

## Supplementary Material

Supplementary Material
