# Supplementary material for: TM9 family proteins control surface targeting of glycine-rich transmembrane domains
Source: J Cell Sci. 2015 Jul 1;128(13):2269–77. doi: 10.1242/jcs.164848 (PMC4510845; doi:10.1242/jcs.164848)
Supplement: Supplementary Material [file supp_128_13_2269__index.html]

TM9 family proteins control surface targeting of glycine-rich transmembrane domains — Supplementary Material 

# TM9 family proteins control surface targeting of glycine-rich transmembrane domains

## JCS164848 Supplementary Material

**Files in this Data Supplement:**

- **Supplementary Material**
