# Supplementary material for: TM9 family proteins control surface targeting of glycine-rich transmembrane domains
Source: J Cell Sci. 2015 Jul 1;128(13):2269–77. doi: 10.1242/jcs.164848 (PMC4510845; doi:10.1242/jcs.164848)
Supplement: Supplementary Material [file supp_128.13.2269_JCS164848.pdf]

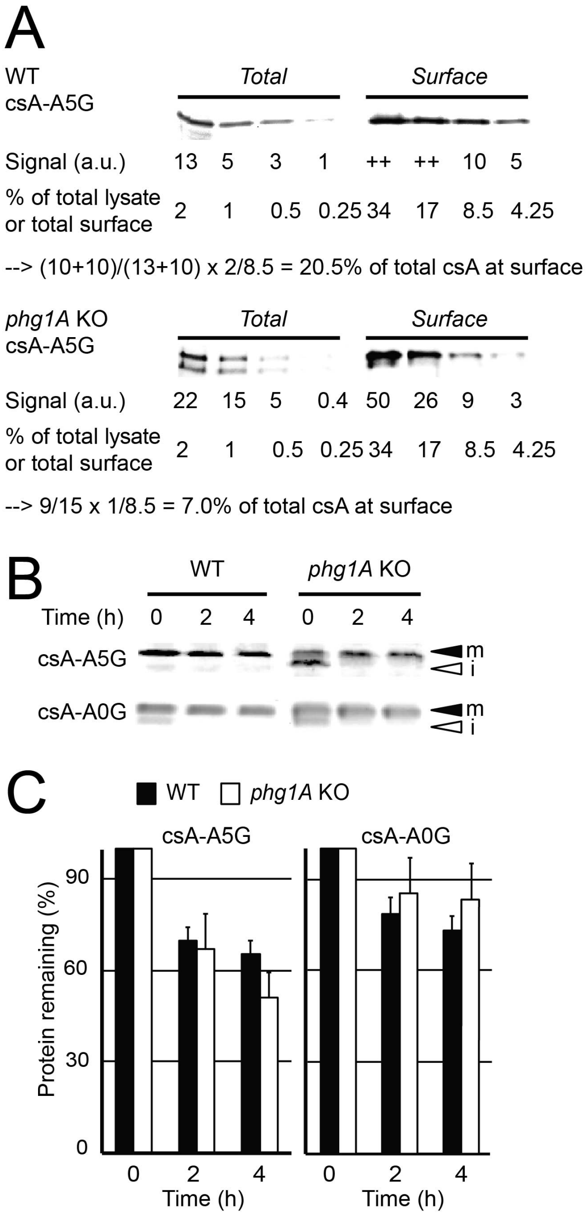

Fig. S1 (Perrin et al.)

Figure S1: Phg1A-dependent targeting of glycine-rich TMDs to the cell surface. (A) Quantification of surface csA fusion protein following surface biotinylation. To determine the percentage of csA protein present at the cell surface, serial dilutions of total or surface proteins were migrated on a SDS-PAGE gel and revealed by Western blot with an antibody recognizing the csA extracellular domain. Only the dilutions

that were in the linear range were compared. The percentages of the total cell lysate (2 to 0.25 %) and of the surface proteins (34 to 4 %) loaded on each lane are indicated. The signal corresponding to each band was quantified using the ImageJ software (http://rsb.info.nih.gov/ij/). To determine the percentage of csA-A5G present at the cell surface, we used the signal corresponding to 4.25 % and 8.5 % of the surface protein, and to 2 % and 1 % of the total protein.

% at the cell surface = $(2\text{xSurface}_{4.25\%} + \text{Surface}_{8.5\%}) / (\text{Total}_{2\%} + 2\text{xTotal}_{1\%})\text{x}2/8.5$

= (2x5+10)/(2x5+13)x2/8.5=20.5 %

(B) csA-A5G and csA-A0G exhibit similar stability in WT and *phg1A* KO cells. Stability of csA-A5G and csA-A0G was assessed in WT and *phg1A* KO *Dictyostelium* cells. To determine the turnover of csA, 5 x 10^6 cells were incubated in HL5 containing 2 mM cycloheximide. Aliquots of 1.5 x 10^6 cells were collected after 0, 2 and 4 hours and resuspended in Sample Buffer (0.103 g/ml sucrose, 50 mM Tris, pH 6.8, 5 mM EDTA, 0.5 mg/ml bromophenol blue, 2 % SDS, 10 % β-mercaptoethanol). Samples were migrated on a 9 % acrylamide gel, and transferred to nitrocellulose using a semi-dry transfer system (Invitrogen, Carlsbad, CA). The membrane was incubated overnight in PBS containing 0.1 % Tween 20 and 7 % milk, then incubated successively with a mouse anti-csA monoclonal antibody (33-294-17) (Bertholdt et al., 1985), and a horseradish peroxidase-coupled goat anti-mouse IgG (Bio-Rad, 1706516). The signal was revealed by ECL. Mature csA has a molecular weight of 80 kDa (arrow m), while the partially glycosylated form has an molecular weight of 68 kDa (arrow i)

(C) The quantification with time of the total amount of csA (mature and immature bands) for csA-A5G and csA-A0G indicates that these two proteins exhibit the same stability in WT and *phg1A* KO cells.

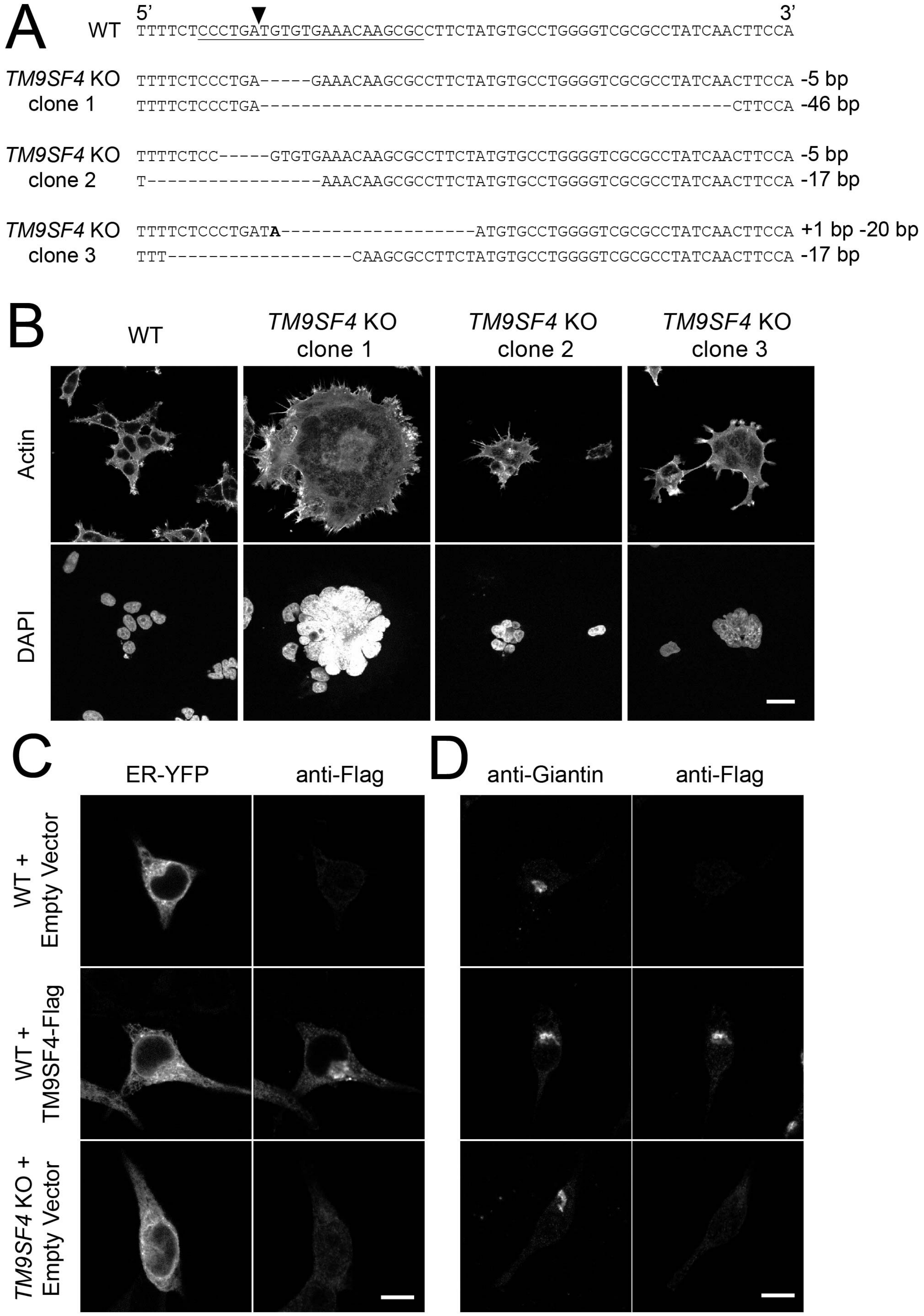

Fig. S2 (Perrin et al.)

Figure S2: Human *TM9SF4* KO cells.

(A) Sequence of *TM9SF4* in human. The sequence targeted by the guide RNA is underlined and the arrow represents the cutting site of the Cas9 nuclease. The sequence of each allele is represented for three independent mutant clones. Deletions are symbolized by (-) and insertions are in bold.

(B) The presence of plurinucleated cells was assessed by actin and nuclei staining. To stain actin and nuclei, HEK293T (WT) or *TM9SF4* KO cells were fixed in PBS containing 4 % paraformaldehyde for 30 minutes, and washed with 20 mM NH_4Cl in PBS. Cells were then permeabilized with PBS containing 0.2 % saponin for 10 minutes and washed in PBS-BSA. Actin was stained with TRITC-labeled phalloidin (Sigma, P-1951) in PBS for 1 hour. Cells were then washed three times with PBS, and nuclei were stained with DAPI (Life Technologies, D1306) in PBS for 5 minutes. Cells were finally washed three times with PBS and mounted in Mowiol. Scale bar: 30 µm.

(C-D) The endoplasmic reticulum and the Golgi apparatus are not affected by the loss or the overexpression of TM9SF4. The endoplasmic reticulum or the Golgi apparatus were revealed using (C) a soluble YFP-KDEL, or (D) an antibody against giantin, in HEK293T cells (WT) (upper panel), in cells overexpressing a Flag-tagged version of TM9SF4 (TM9SF4-Flag, intermediate panel), or in *TM9SF4 KO* cells (lower panel). Scale bar: 10 µm.

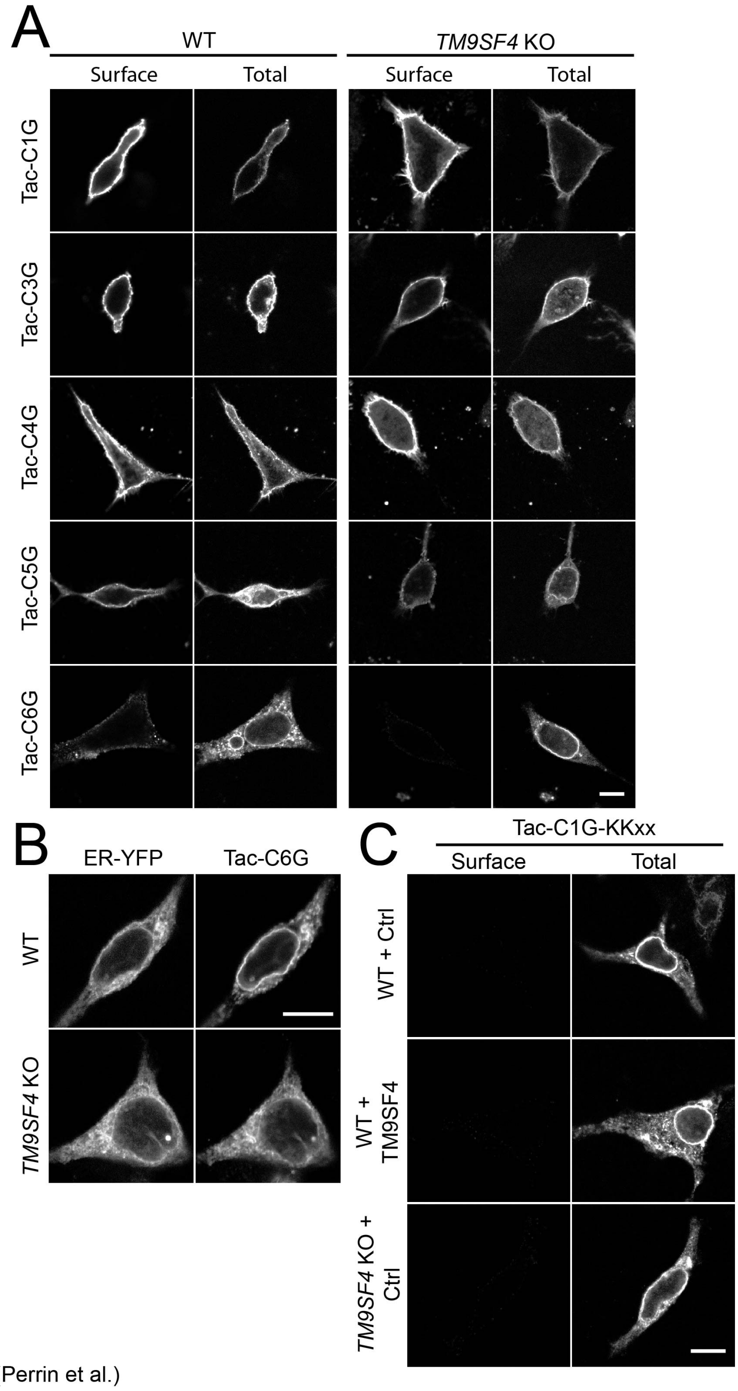

Fig. S3 (Perrin et al.)

Figure S3: TM9SF4 ensures specifically localization of glycine-rich TMDs to the cell surface.

(A) Multiple glycine residues in the TMD of Tac proteins ensure its localization in the endoplasmic reticulum in human cells. Tac chimeric proteins with 1, 3, 4, 5 or 6 glycine residues in their TMD were expressed in HEK293T cells (WT) or in *TM9SF4*

KO cells. The Tac fusion proteins were labeled before (Surface) or after (Total) cell permeabilization.

(B) The majority of Tac-C6G colocalizes with a co-expressed ER-localized soluble YFP-KDEL in WT (upper panel) or *TM9SF4* KO cells (lower panel). Scale bar: 10 µm.

(C) Loss or overexpression of TM9SF4 does not affect dilysine-mediated ER retention. Tac-C1G-KKxx was expressed in WT cells (upper panel), WT cells overexpressing TM9SF4 (intermediate panel) or in *TM9SF4* KO cells (lower panel). The Tac fusion protein was labeled before (Surface) or after (Total) cell permeabilization. Scale bar: 10 µm.

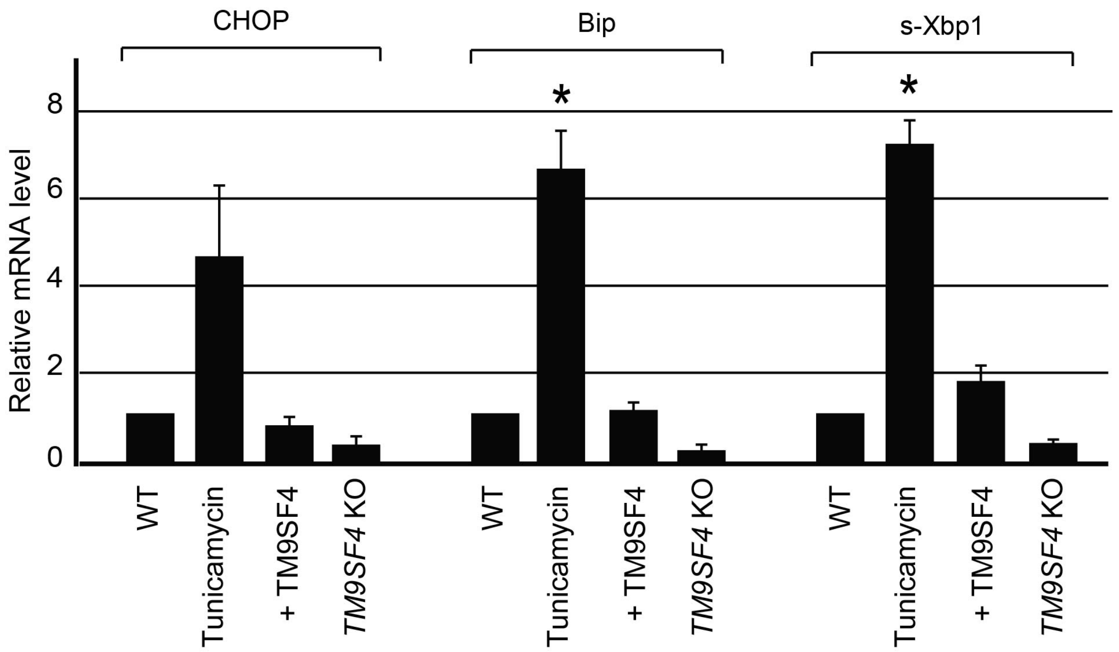

Fig. S4 (Perrin et al.)

Figure S4: ER stress response genes are not induced in human cells when TM9SF4 is lost or overexpressed.

Total RNAs from HEK293T human cells were extracted (RNeasy Mini kit, Qiagen). RT-PCR was realized with 1µg of total RNAs (Qscript cDNA synthesis, Quanta Biosciences). Analysis of mRNA levels of CHOP, Bip and spliced-Xbp1 (s-Xbp1) was realized by real-time quantitative PCR (StepOne System, Life Technologies). The primers used are described in (Oslowski and Urano, 2011). Cells treated with Tunicamycin (5 µg/ml) during 5 hours were used as positive control. The average and SEM of at least three independent experiments are indicated. * indicates a significant difference (Student *t* test $p < 0.01$).
